# Quality of Health Information on the Internet for Prostate Cancer

**DOI:** 10.1155/2018/6705152

**Published:** 2018-12-04

**Authors:** Dwayne T. S. Chang, Robert Abouassaly, Nathan Lawrentschuk

**Affiliations:** ^1^Department of Urology, Sir Charles Gairdner Hospital, WA, Australia; ^2^Urology Institute, University Hospitals Case Medical Center, Cleveland, OH, USA; ^3^Department of Surgery, Division of Urology, Louis Stokes Cleveland VA Medical Center, Cleveland, OH, USA; ^4^Department of Urology, Austin Hospital, VIC, Australia; ^5^Olivia Newton-John Cancer Research Institute, Austin Hospital, VIC, Australia

## Abstract

**Introduction:**

To compare (1) the quality of prostate cancer health information on the Internet, (2) the difference in quality between websites appearing earlier or later in the search, and (3) the sources of sponsorship for each of these websites.

**Materials and methods:**

The top 150 listed websites on the Google search engine for each of the 11 search terms related to prostate cancer were analysed. Quality was assessed on whether the website conforms to the principles of the Health On the Net Foundation. Each of these websites was then reviewed to determine the main source of sponsorship. Statistical analysis was performed to determine if the proportion of HON accreditation varied among the different cohorts of listed websites and among the 11 search terms used.

**Results:**

In total, 1650 websites were analysed. Among these, 10.5% websites were HON-accredited. The proportion of HON-accredited websites for individual search terms ranged from 3.3% to 19.3%. In comparison with the search term of “Prostate cancer,” four search terms had statistically significant odds ratio of the rate of HON accreditation. Websites 51–150 were statistically less likely to have HON accreditation than websites 1–50. The top three website sponsors were journal/universities (28.8%), commercial (28.1%), and physician/surgeon (26.9%).

**Conclusions:**

The lack of validated and unbiased websites for prostate cancer is concerning especially with increasing use of the Internet for health information. Websites sponsored or managed by the government and national departments were most likely to provide impartial health information for prostate cancer. We need to help our patients identify valid and unbiased online health resources.

## 1. Introduction

Prostate cancer remains a huge burden in Australia and the United States of America (USA). In 2012, the age-standardised incidence rate of prostate cancer in Australia was 162.7 per 100,000 males, an increase from 137.4 per 100,000 in 2002 [1]. Australia also has a higher age-standardised incidence rate of prostate cancer than the World Health Organisation (WHO) world standard population and the USA in 2012 (162.7 vs. 119.5 vs. 108.4 per 100,000 males, respectively) [1, 2]. In the same period of time, the risk of diagnosis of prostate cancer rose from 1 in 10 to 1 in 7 in men before the age of 75 years [1]. Currently, more men are getting diagnosed with prostate cancer, so more will be seeking answers about their disease and the varied treatment options.

Patients are increasingly resorting to the Internet for medical information. An Australian study of nearly 3000 patients found that 63% of them accessed the Internet within the previous month with 28% seeking health information online and 17% obtained information in relation to medical conditions managed by their family practitioner at the time [3]. In that study, patients within the age group of 45–64 years were the second most likely cohort to obtain health information online, whereas those in the 65–74 years cohort were the fourth most likely [3]. In 2012, the highest incidence of prostate cancer diagnosis was in the 60–69 years age group [1]. A survey by Pai et al. on men with prostate cancer showed that not only were most of them knowledgeable with using the Internet, most men and their spouses/partners want to be able to access their health information over the Internet [4]. Better diagnostic tests have led to younger men being diagnosed with prostate cancer, and these men are more likely to use the Internet to obtain health information.

It is important to keep a level head when browsing through the ocean of health information on the Internet. Anyone in the world can easily set up a website and publish any form of data to be accessed by the mass of unknowing Internet users. The amount of unregulated and potentially biased information online may end up confusing lay users of the Internet [5]. Fortunately, services are available to direct Internet users towards trustworthy online health information. One of the earliest examples is the Health On the Net (HON) Foundation, which is a nonprofit and nongovernmental organisation found in 1995 from a collaboration of the WHO and prominent physicians and scientists all over the world [6]. This organisation certifies websites that provide objective and transparent health information and is currently one of the most widely accepted certification tools in use [6].

Our objectives in this study were to compare (1) the quality of health information on the Internet relating to prostate cancer, (2) the difference in quality between websites appearing earlier or later in the search, and (3) the sources of sponsorship for each of these websites.

## 2. Materials and Methods

We used a tried and tested methodology as previously described in other studies [7–10]. The Google search engine (http://www.google.com) was used to search for 11 keywords related to prostate cancer. These keywords were “Prostate cancer;” “Prostate specific antigen” (PSA); “Transrectal prostate biopsy;” “Transperineal prostate biopsy,” “MRI prostate;” “Prostate-specific membrane antigen” (PSMA); “Radical prostatectomy;” “Robotic prostatectomy;” “Prostate radiotherapy;” “Prostate chemotherapy;” and “Prostate cancer hormone therapy.” Throughout this entire study, “sponsored links” presented by the Google search engine anywhere on the search page or under a banner were not included in the list of websites analysed.

Sponsorship of each website was analysed and categorised in a similar way to previous studies on the quality of health information on the Internet [7–9]. These categories were (i) journal/university institutions (e.g., scientific journals and University of Oxford), (ii) nonprofit organisations (e.g., Prostate Cancer Foundation of Australia and Cancer Council Australia), (iii) governmental/national institutions (e.g., Healthdirect Australia and World Health Organisation), (iv) commercial (e.g., da Vinci Surgery and GlaxoSmithKline), (v) physician/surgeon (including their corresponding professional organisations, e.g., American Urological Association), (vi) nondoctor health professionals (such as naturopaths), and (vii) others (e.g., personal blogs with no other affiliations). If the source of sponsorship was not obviously apparent, such as a website with multiple types of sponsors, the website was explored by all authors until the primary source of sponsorship could be determined.

The first 150 websites found from each search were selected to be analysed. The HON Foundation's HONcode web browser toolbar (available from http://www.hon.ch/) was used on a personal computer. An indicator on the toolbar lights up automatically if the website viewed is currently accredited by the HON Foundation. The HONcode toolbar was used in several studies and was found to be a valid and reliable tool [7–10].

For quality control, nonaccredited websites found for the search term “Prostate cancer” were individually evaluated to determine HON Foundation principles [11] were adhered to and then compared to the findings from using the automated HONcode web browser toolbar. The HON Foundation reviewed each website according to their published principles [11] which includes authority of the authors, complementarity of the information, privacy of submitted personal data, attribution of information to sources, justifiability of information, transparency, financial disclosure, and advertising policy of websites.

The first 150 websites found for each term were divided into tertiles (first, middle, and last 50 search results). The proportion of HON-accredited websites within each tertile was analysed and compared using the chi-squared test. This analysis determines whether HON-accredited websites were more likely to appear in the first, middle, or last tertile of search results. The proportions of accredited websites were compared across search terms and languages using the chi-squared test (or Fisher exact tests when cell counts were less than five). All statistical tests were two-sided, and statistical significance was defined as *P* < 0.05. Univariate logistic regression analysis was performed using the variables of search terms and tertiles of search results. The referent group for search terms was the word “prostate cancer” as it was the base search term. The referent group for tertiles 2 and 3 was tertile 1 (the first 50 websites) as it had the highest proportion of HON-accredited websites. Odds ratios and 95% confidence intervals were calculated from the logistic regression analysis. Analyses were performed using SAS 9.3 (SAS Institute Inc., Cary, NC, USA).

## 3. Results

**Figure 1:**
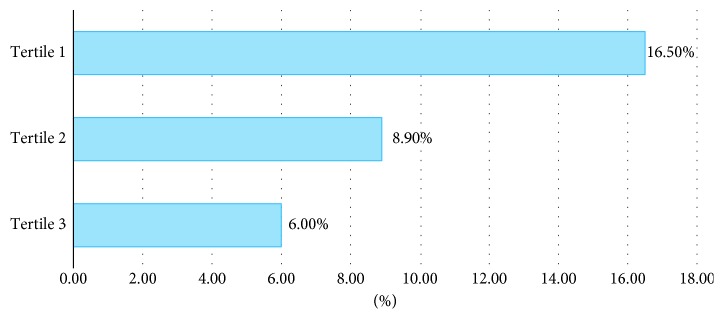
Rates of HON-accredited websites within each tertile group. *Note.* Tertile 1, websites 1–50; Tertile 2, websites 51–100; Tertile 3, websites 101–150.

**Figure 2:**
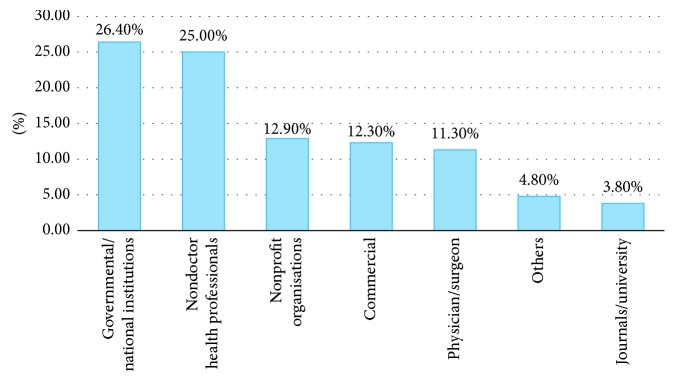
Rates of HON accreditation for various types of sponsorship.

In total, 1650 websites were analysed. Among these, 173 (10.5%) websites were HON-accredited. Less than 10% of search results were made up of HON-accredited websites in six out of the 11 searched terms. The proportion of HON-accredited websites for individual search terms ranged from 3.3% (robotic prostatectomy) to 19.3% (prostate cancer hormone therapy) (Table 1). In comparison with the search term of “Prostate cancer”, four search terms had statistically significant odds ratio of the rate of HON accreditation (Table 2). The proportion of HON-accredited websites decreased progressively from tertiles 1 to 3 (Figure 1). Websites in tertiles 2 and 3 were more likely to have lower rates of HON accreditation than websites in tertile 1, and this achieved statistical significance (Table 2). The rates of website sponsors in descending frequency were journal/universities (28.8%), commercial (28.1%), physician/surgeon (26.9%), nonprofit organisations (8.0%), governmental/national institutions (6.7%), others (1.27%), and nondoctor health professionals (0.2%). Among the various sources of sponsorship, websites sponsored by governmental/national department sources had the highest rate of HON accreditation (26.4%) followed by those sponsored by nondoctor health professionals (25.0%), nonprofit organisations (12.9%), and commercial sources (12.3%) (Figure 2). Although websites sponsored by nondoctor health professionals ranked highly, there were a total of only four of such websites found in this search, and only one was HON-accredited. The number of HON-accredited websites found by manually applying the HON Foundation principles correlated with the number found via using the automated HONcode web browser toolbar.

## 4. Discussion

A staggering finding was that even after accounting for overlap of websites that are relevant with more than one search term, there were at least more than 21 million websites relating to prostate cancer (Table 1). It is difficult to imagine that a lay person would be able to keep a level head and critically appraise each website with the vast amount of unfiltered information available. A silver lining is that the proportion of HON-accredited websites is slowly growing. In a similar study done previously [8], the number of HON-accredited websites among the first 150 listed in the search term “prostate cancer” was 13 in 2004, 18 in 2009, and 23 in this study in 2016. This shows a steady growth of approximately one extra HON-accredited website each year in the past 12 years in the first 150 listed websites for the search term “prostate cancer.”

Overall, less than one in nine websites found in this study had HON accreditation. Such a low proportion can make it difficult for our patients to safely delineate fact from fiction on these websites and may promote distrust by physicians of cancer resources from the Internet. Previous studies in 2004–2010 found the rate of HON accreditation to range from 10% to 29% [8, 9]. It is interesting that the overall proportion of HON-accredited websites have lessened. Theories for this include growth of new unaccredited websites outpacing the accredited ones, “drop-out” or discontinuation of previously HON-accredited websites, and unaccredited websites achieving higher user traffic than accredited websites. Furthermore, four search terms had statistically different odds ratio of HON accreditation. This means selecting the right search term can result in a list of websites with a greater proportion of HON accreditation, and vice versa.

A reassuring finding was that more than a quarter of all websites sponsored by governmental/national institution sources were accredited by the HON Foundation. Although websites sponsored by commercial sources accounted for more than a quarter of all websites in this study, the rate of HON accreditation among them ranked fourth (less than one in eight). Interestingly, the same applied to websites sponsored by journals/universities which were slightly more common than commercial-type websites but fared much worse in the rate of HON accreditation (less than one in 25 websites). As governmental/national department and journal/university sponsors typically have strong financial backing, the reason for the lower rate of HON accreditation with journal/university-sponsored websites may be related to being unaware of this service and its associated benefits rather than financial cost.

In this study, there were nearly as many commercial websites as journal/university sources, which was the most prevalent. This can be disadvantageous to us as doctors as commercial websites have just as much exposure to the general public on the online search. It is then up to the patient to decide which website to read, and branding of websites may influence this process as readers may opt for articles by familiar sources. It was demonstrated that the general public in Australia were more familiar with simpler brands containing the words, “general practitioner,” “research,” and “prostate cancer,” instead of the one containing “urological” [12]. They also had limited understanding of the role of urologists in prostate cancer and the central organisation that Australian urologists belong to [12]. There may be a role for adding further clarity to the brand names of national urological associations to illustrate the key services we provide [12]. We as doctors should also act as custodians of evidence-based knowledge. One such custodian for urologists is the BJU International journal which centralises quality evidence-based urological knowledge. For example, this journal has key papers in the myriad of localised and systemic [13] treatment of prostate cancer, such as active surveillance [14], robotic prostatectomy [15], brachytherapy [16], and even alternative treatments like phytotherapy [17]. In the same way, reputable organisations can summarise evidence-based knowledge into a centralised online library in simple language so that our patients do not have to sift through the ocean of unsorted information on the Internet.

We used the Google search engine as it is the most widely used search engine; therefore, it is the one most likely to be used by our patients for whom we conducted this study for. There are other Internet search engine services available. Each of them uses different mechanics or algorithms to search for relevant websites; thus, the order of listed websites may differ even if the same study methodology is performed using a different search engine. In addition, specific alterations can be made to the website structure and content to artificially increase the relevance factor in the eye of the search engine thus placing it higher up on the list of search results. Furthermore, the search engine or Internet browser may analyse the history of previous Internet searches on the device and customise future search results to match it. To minimise this effect, we deleted all website and Internet browser information cookies and data cache prior to conducting the Internet search. However, not all patients may take this extra precaution routinely and may end up with search results influenced by their previous use of the search engine and Internet browser. For example, if the user had a significant history of using the device for online shopping, their search for websites relating to prostate cancer may favour commercially oriented websites instead of educational ones.

Several non-modifiable limitations were present in this study. The order of website listing on a search engine result depends on the real-time popularity of each website, and this may change on a daily basis. Theoretically, it is possible for a website ranked 99 today to be ranked 101 tomorrow thus causing movement of websites between tertile groups. There may also be a risk of false negatives as not all websites without HON accreditation provide biased or invalid information. This may simply be due to lack of awareness of the existence of this service or the financial cost of maintaining HON certification. HON accreditation is free of charge for the first year but subsequent to that there is an annual membership fee to renew and extend the HON certification. The type and popularity of each website will determine the amount of fee charged, which usually ranges between EUR50 and EUR325 per year [18]. However, renewal of HON accreditation does not automatically happen after payment of membership fee; essentially, there is no blind renewal of HON certification. Every year, each member website will be reviewed by a team from the HON Foundation and if they find that the website has deviated from the HON principles, the payment will be refunded and certification revoked [18].

To readers from the general population, we advise to use general and less-complicated search terms as from our experience, this may allow the search engine to present a better representation of relevant websites. For example, searching for “prostate cancer test” will result in a lot more search results than searching for “prostate cancer PSA, MRI scan” as websites with PSA-only or MRI-only information may not be listed higher on the search even if they happen to provide valid information. We also advise to select the first 50 websites of the search which, in our study, has a higher proportion of websites with unbiased information. It is also a good idea to focus on websites by educational institutions, governmental organisations, and scientific journals which tend to be unbiased. Websites by doctors need to be critically assessed to determine as some are meant to promote or publicise their services and others have the sincere aim of educating the general public. Another important tip is that most websites have an “About us/this organisation” webpage which is key to finding out whether these websites hold potential bias due to their sponsorship source or partnership.

## 5. Conclusions

The lack of validated and unbiased websites for prostate cancer is concerning especially with the rise in Internet use for health information by patients and physicians alike. Although there was slow growth in the number of HON-accredited websites in the main search term of “prostate cancer,” the overall proportion of HON-accredited websites was still very low. Websites sponsored or managed by the government and national departments were the most likely to provide impartial health information for prostate cancer. We can help our patients identify valid and unbiased health resources. We need to act as custodians of valid information by creating online libraries of valid information or direct our patients to these sources.

## Figures and Tables

**Table 1:** Rates of HON-accredited websites among the search terms.

Search term	Total websites	HON+	HON−	%HON+ (%)
Prostate cancer	21700000	23	127	15.3
Prostate specific antigen	1580000	23	127	15.3
Transrectal prostate biopsy	177000	12	138	8.0
Transperineal prostate biopsy	38900	14	136	9.3
MRI prostate	7710000	9	141	6.0
Prostate-specific membrane antigen	835000	6	144	4.0
Radical prostatectomy	687000	18	132	12.0
Robotic prostatectomy	437000	5	145	3.3
Prostate radiotherapy	2260000	14	136	9.3
Prostate chemotherapy	23100000	20	130	13.3
Prostate cancer hormone therapy	1710000	29	121	19.3

*Note.* HON+, HON-accredited; HON−, non-HON-accredited; %HON+, percentage of HON-accredited websites out of a total of 150 websites for each search term.

**Table 2:** Univariate logistic regression analysis showing odds ratio estimates comparing search terms to “Prostate cancer” and tertiles 2 and 3 against tertile 1.

Odds ratio comparison	Point estimate	95% confidence interval limits	
PSA vs. Prostate cancer	1.00	0.53	1.89
^*∗*^Transrectal prostate biopsy vs. Prostate cancer	2.12	1.01	4.47
Transperineal prostate biopsy vs. Prostate cancer	1.79	0.87	3.65
^*∗*^MRI prostate vs. Prostate cancer	2.90	1.29	6.56
^*∗*^PSMA vs. Prostate cancer	4.47	1.75	11.40
Radical prostatectomy vs. Prostate cancer	1.34	0.68	2.62
^*∗*^Robotic prostatectomy vs. Prostate cancer	5.41	1.98	14.73
Prostate radiotherapy vs. Prostate cancer	1.79	0.87	3.65
Prostate chemotherapy vs. Prostate cancer	1.18	0.61	2.28
Prostate cancer hormone therapy vs. Prostate cancer	0.75	0.41	1.38
^*∗*^Tertile 2 vs. tertile 1	2.04	1.36	3.07
^*∗*^Tertile 3 vs. tertile 1	3.22	2.03	5.11

*Note.* ^*∗*^Statistically significant difference (95% confidence interval not crossing the value of 1).

## Data Availability

The data used to support the results of this study are available on the publicly accessible and searchable World Wide Web.
